# Talks on Obstetrics

**Published:** 1918-02

**Authors:** 


### BOOK REVIEWS

Books mentioned may be inspected at and ordered through this office. So far as possible, books received in any month will be reviewed in the issue of the second month following. Pamphlets, quarterly and similar periodicals, reports, transactions, etc., will, as a rule, merely be mentioned.

---

**Talks on Obstetrics,** by Rae Thornton La Vake, M. D., Instructor in Obstetrics and Gynecology, University of Minnesota; Obstetrician in charge of the Out-Patient Obstetric Department of the University of Minnesota; Associate Attending Obstetrician and Gynecologist to the Minneapolis City Hospital; Obstetrician in charge of the Out-Patient Obstetric Department of the Wells Memorial Dispensary; Obstetrician to the Swedish and Abbott Hospitals, Minneapolis; one time Assistant Resident Obstetrician to the Sloan Hospital for Women in New York. Cloth, 157

pages. Price $1.00. St. Louis, G. V. Mosby Company, 1917.

The author addresses himself to students and practitioners to whom he imparts in an easy, familiar style, a few precepts and observations calculated "to give a perspective that will help to establish the relative importance of certain problems." This is not a text book nor a substitute therefor. The author has done his work acceptably, giving his readers the benefit of his wide experience and advising them sanely and conservatively. The fourteen chapters cover concisely all the complications and problems that the average physician is likely to meet in his practice of obstetrics. The book is well indexed.—F. W. B.

